# Development of the Japanese version of the Other As Shamer Scale using item response theory

**DOI:** 10.1186/s13104-020-05027-z

**Published:** 2020-04-05

**Authors:** Yoichi Hiramatsu, Kenichi Asano, Yasuhiro Kotera, Toshihiko Sensui, Ayumu Endo, Eiji Shimizu, Jaskaran Basran, Ken Goss

**Affiliations:** 1 Research Center for Child Mental Development, Chiba University Graduate School of Medicine, 1-8-1 Inohana, Chuo-ku, Chiba, 260-8670 Japan; 2 The Japanese Centre for Compassionate Mind Reserch and Training, Tokyo, Japan; 3 Komachi Clinical Psychology Office, 2-11-1, Minamisaiwai, Nisi-ku, Yokohama-MSBldg, Yokohama, Kanagawa, 220-0005 Japan; 4 Department of Psychological Counseling, Faculty of psychology, Mejiro University, 4-31-1 Nakaochiai, Shinjyuku-ku, Tokyo, 161-0032 Japan; 5 Human Sciences Research Centre, University of Darby, Kedleston Road, Derby, DE22 1GB UK; 6 Department of Psychology, Faculty of Human Studies, Saitamagakuen University, 1510, Kizoro, Kawaguchi, Saitama 333-0831 Japan; 7 Department of Psychology, Faculty of Letters, Komazawa University, 1-23-1, Komazawa, Setagaya-ku, Tokyo 154-8525 Japan; 8 Department of Cognitive Behavioral Physiology, Graduate School of Medicine, 1-8-1 Inohana, Chuo-ku, Chiba, 260-8670 Japan; 9 Centre for Compassion Research and Training,College of Health and Social Care Research Centre, University of Derby, Derby, DE22 1GB UK; 10 The Compassionate Mind Foundation, Office 29, Riverside Chambers, Full Street, Derby, DE1 3AF UK; 11 Coventry & Warwickshire Partnership Trust, Coventry Eating Disorder Service, Swanswell Point, Stoney Stanton Road, Coventry, CV1 4FS UK

**Keywords:** Shame, External shame, Item response theory

## Abstract

**Objective:**

External shame reflects a person’s anxiety that he or she might be rejected by others. The Other as Shamer Scale (OAS) is a scale for assessing external shame. The Japanese version of the OAS was developed, and its reliability and validity were examined using Item Response Theory (IRT).

**Results:**

A survey was conducted with university students (N = 199). Exploratory factor analysis of the results indicated a significantly high factor loading on the first factor, which was identical to the original version of the scale as well as high internal consistency. Moreover, the results confirmed that each item had adequate discrimination and information levels, suggesting that external shame could be discriminated against with high accuracy for a wide range of relatively low and relatively high external shame groups. These results suggest that the OAS could be used to screen external shame as a stress factor and to assess intervention effects.

## Introduction

Shame can be a social event (e.g., being judged and shamed in the eyes of others) or a private feeling linked to our judgments of our feelings, ability to fantasize, and characteristics. Shame can guide behavior, influence feelings about ourselves, and shape our sense of self-identity and feelings about our social acceptability and desirability [1–3].

Correlations have been found between shame and many psychiatric symptoms, such as borderline personality disorders [4], eating disorders [5, 6], anxiety [7], depression [3, 8, 9], and paranoia [10], among others. Therefore, shame is an essential factor related to mental health.

According to Gilbert [11], two types of shame exist. One is “Internal shame,” which is related to the internal dynamics of the self and how the self judges and feels about itself [11]. Internal shame relates to the tendency to attend to negative aspects of the self and to maintain global self-judgments of the self as bad, inferior, and flawed [1–3]. The other type is “external shame,” which is associated with the following tendencies: being worried that others would see the self as uninteresting or boring and, thus, the self would be rejected or excluded from valuable relationships [11]. External shame has been defined as shame that arises primarily from the process of being shamed by others, which is the source of this type of shame [1, 11].

External shame is caused by the consciousness of others, that is, the concept that the self is negatively evaluated by others, which is correlated with depression [12], one’s body image related to eating disorders [6], and one’s self-image related to social anxiety disorders [13]. However, in Japan, no standardized scales exist to measure external shame.

Moreover, the suggestion has been made that most stress response scales in Japan were developed based on classical test theory [14]. Classical test theory has a significant problem that survey results are highly affected by the characteristics and quality of the sample because statistics are defined based on the population [14]. Item response theory (IRT) is a paradigm for solving this problem. Different from the reliability coefficient that previously assessed the mean accuracy of an entire scale, IRT measurement accuracy is expressed as a function of characteristic values on a continuous scale that indicates latent traits (θ) and a point at which a measurement value with high accuracy is indicated about the entire test as well as being based on each item. Therefore, the appropriateness of each item can be judged from the perspective of the measurement purpose of the test [15]. Moreover, the practical utility of the scale can be examined from diverse perspectives.

Based on this information, this study aims to explore the development of the Japanese version of the OAS to assess trait shame—especially external shame. The reliability and validity of the OAS were also examined, and its measurement accuracy was examined using the IRT.

## Main text

### Participants

Responses were collected from university students (N = 205). Most of the students were majoring in psychology, and some were majoring in another course of study. Data on 199 participants were used for the analysis after excluding six participants who did not respond to all or a part of the questions. Among the 199 participants, 130 (65%) were women, and two had an unknown gender. The age range of the participants was 18 to 36 years (M = 19.68; SD = 1.62).

### Measures

#### Japanese version of Other As Shamer Scale (OAS)

OAS is a self-report instrument composed of 18 items that assess external shame Goss et al. [16]. Respondents were asked to indicate the frequency of their feelings and experiences related to each item on a five-point scale ranging from 0 (*Never*) to 4 (*Almost Always*).

After receiving approval of the original authors, two experts, including the author, translated the original version of the scale into Japanese. Two native English speakers from Crimson Interactive Japan Co., Ltd. conducted the back translation, and the translated sentences were compared with the original English, which indicated differences in the meaning of certain items. Therefore, the Japanese translation was revised, and the back-translation was repeated, which confirmed no differences in the meaning between the original and the translated versions of the scale. The English version of the scale resulting from the back translation was sent to the two original authors, one of whom pointed out differences in the meaning of specific items. After several discussions with the authors, the Japanese version of the scale was revised according to the advice of the original authors, who indicated sufficient consistency of the scale. Finally, the Japanese version of the OAS was developed.

#### Japanese version of Beck Depression Inventory-II (BDI-II)

Construct validity of the Japanese version of the OAS was examined based on the correlation with depressive tendencies using the Beck Depression Inventory-II (BDI-II) developed by Beck et al. [17]. The Japanese version of BDI-II was developed by Kojima et al. [18] and demonstrates a high degree of validity and reliability as an assessment scale of depression.

As previously described, previous studies have repeatedly demonstrated correlations between the OAS and depressive symptoms. Allan et al. [19] examined correlations between external shame and depressive tendencies based on correlations between OAS and the Beck Depression Inventory (BDI), which indicated a high positive correlation (r = 0.58 to 0.73). This study was also expected to demonstrate a correlation between OAS and BDI-II, similar to Allan et al. [19].

### Procedures

A survey was conducted at three universities in Japan during December 2019. Participants for the present study were university students taking a psychology course. Because the survey was conducted in a psychology classroom, some students were majoring in other subjects. The survey conducted anonymously for ethical reasons. Written explanations were provided in advance to participants to describe the purpose of the survey, the protection of their personal information, and the voluntary nature of their participation. Participants’ response to the survey was regarded as their consent for participation. This study was conducted after obtaining the approval of the ethics committee of Chiba University (No. 3441).

Data analysis was conducted using SPSS Statistics 26. The Graded Response Model (GRM), which is applicable to multi-item tests, was employed in the IRT analysis because OAS uses a five-point scale. EasyEstGRM [20] was used for the calculation. We used D = − 1.7 as the scale factor for calculating the discrimination parameters.

### Results

First, confirmatory factor analysis was conducted using the principal factor method, which indicates that the contribution of the first factor was 52.86% (eigenvalue = 9.72), the second factor was 6.78% (eigenvalue = 1.22), and the third factor was 5.73% (eigenvalue = 1.03). These results confirmed that that scale had a one-factor structure based on the high contribution ratio of the first factor and the differences in eigenvalues. Moreover, as a measure of reliability, Cronbach’s α, which was 0.942, indicated a significantly high internal consistency that was identical to the original version of the scale.

Next, the construct validity of the scale was examined through a correlation analysis between OAS and BDI-II, which indicated a high positive correlation (r = 0.57, p < 0.001) similar to Allan et al. [19].

When conducting the IRT analysis, point-biserial correlation coefficients were calculated, and a one-factor factor analysis was conducted using polychoric correlation coefficients. The results indicated a range of point-biserial correlation coefficients from 0.53 to 0.84, which suggests a strong positive correlation. Moreover, the results of the one-factor factor analysis using polychoric correlation coefficients indicated that the eigenvalue of the first factor was 10.53, of the second factor was 1.26, and of the third factor was 1.06. The differences in the eigenvalues suggested a one-factor structure for the scale.

**Fig. 1:**
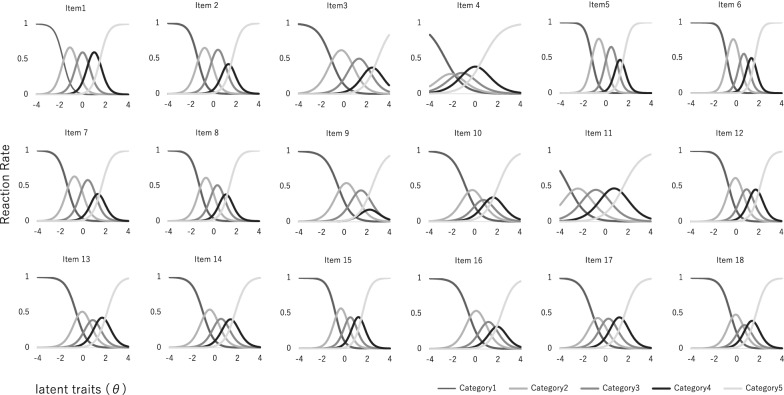
Category response curve of each item

**Fig. 2:**
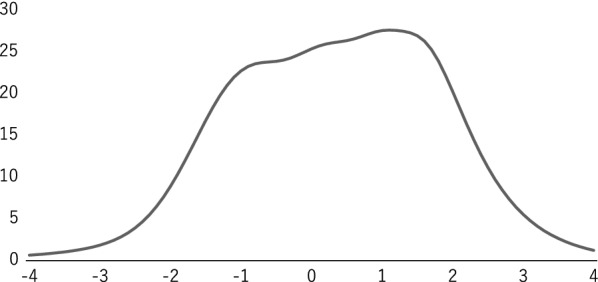
Test information curve of all items

Table 1 provides the results of calculating the discrimination and difficulty parameters of each item using GRM. Figure 1 shows the category response curve of each item, and Fig. 2 shows the test information curve of the entire scale. The mean discrimination parameter is observed to be 1.3 (0.70–2.0) with no extreme dispersion, although Item 4 (0.70) and Item 11 (0.73) had relatively low values compared with the other items. These results indicated that each item had middle or very high discrimination. The difficulty parameters did not show a significant deviation; b1: − 0.52 to − 0.3.3, b2: 0.73 to − 1.6, b3: 2.1 to − 0.65, and b4: 3.0 to 0.71. Moreover, only b1 of Item 11 was relatively high, at − 3.3, suggesting a strong tendency to respond when the item was applicable.

**Table 1:** Item parameters using the graded response model

Item	Item parameters				
	*A*	*B1*	*B2*	*B3*	*B4*
Item1	1.60	− 1.68	− 0.49	0.53	1.54
Item2	1.51	− 1.38	− 0.17	0.98	1.68
Item3	0.96	− 1.12	0.67	2.02	3.00
Item4	0.70	− 2.64	− 1.67	− 0.65	0.71
Item5	1.95	− 1.24	− 0.01	0.94	1.56
Item6	2.07	− 0.85	0.30	1.02	1.64
Item7	1.41	− 1.38	− 0.13	0.98	1.66
Item8	1.49	− 1.26	− 0.12	0.76	1.39
Item9	1.00	− 0.52	0.93	2.05	2.46
Item10	0.94	− 0.80	0.40	1.19	2.08
Item11	0.73	− 3.25	− 1.61	− 0.05	1.60
Item12	1.44	− 0.65	0.52	1.33	2.12
Item13	1.23	− 0.60	0.47	1.26	2.12
Item14	1.21	− 1.04	0.13	0.98	1.80
Item15	1.58	− 0.81	0.15	0.86	1.58
Item16	1.09	− 0.57	0.73	1.60	2.31
Item17	1.13	− 1.17	− 0.21	0.73	1.72
Item18	1.33	− 0.55	0.37	0.98	1.71

The test information curve expresses a trapezoid shape confirming that the amount of information was relatively high for the range of latent trait values from − 1.8 (error = 0.29) to 2.4 (error = 0.28), shifting toward the X-axis in a positive direction. The maximum value of the test information was 27.6 (error = 0.19), which was achieved when the latent trait value was 1.1.

### Discussion

The results of the IRT analysis that examined the measurement accuracy of the scale indicated that the discrimination parameter of each item was included in the range from 0.70 to 2.0, or no extreme dispersion. However, Item 4 (0.70) and Item 11 (0.73) had relatively low values compared with the other items. According to the discrimination parameter criteria of Baker [21], Items 4 and 11 could be included in the “middle level,” and the other items could be included in “high” or “very high” levels. Therefore, the discrimination ability of the scale was considered to have reached a sufficiently adequate level. In the original version of the scale, the contribution of Item 11 was also relatively low. Additionally, the difficulty parameters of Items 4 and 11 shifted toward the X-axis in a negative direction relative to the other items, suggesting that the participants tended to score high on these items. Item 4 consisted of the statement, “I feel insecure about other’s opinions of me,” which was different from the other items because this item does not inquire into others’ negative evaluations. Although Item 4 is related to external shame, it might include a broader meaning of the word shame. Arimitsu [22] suggested that the functions of the sense of guilt and shame between Japanese and European or American people reflected no fundamental differences. In contrast, Matsui et al. [23] suggested that Japanese junior and senior high school students had a higher “sense of shame about conformity with others,” that is, concern about being different from others, relative to Turkish junior and senior high school students. Cultural differences might have affected the responses to Item 4.

### Conclusions

This study indicated that each item of the Japanese version of the OAS has appropriate discrimination ability and information and could discriminate at high accuracy external shame in the range from relatively low to moderately high. Therefore, using this scale as a screening test of external shame is possible, as is examining the effects of interventions for depression, among other disorders.

## Limitations

A limitation of the present study is that the test–retest reliability was not investigated. One suggestion is that future studies should examine the test–retest reliability of the scale to confirm its stability. Additionally, the construct validity of the scale was not sufficiently examined in this study.

In this study, we conducted a survey with a relatively small number of students at three universities. A larger, randomized survey is required in future studies. In addition, only the gender, age, and major of a participant were obtained; collecting background information such as economic status in greater detail is necessary. The results of this study should be interpreted with caution considering the lack of information on participants’ economic status.

Previous studies indicated correlations between external shame and depression, as well as various factors that aggravate mental health, such as “anger.” Future research should investigate whether the same correlations can be observed in the Japanese version of the OAS.

## Data Availability

The datasets generated or analyzed during the current study are available from the corresponding author on reasonable request.

## Abbreviations

OAS: Other as Shamer Scale
IRT: Item response theory
BDI-II: Beck depression inventory-II
GRM: Graded response model
